# The Impact of Orthodontic Treatment on Body Weight Due to Change in Dietary Habits

**DOI:** 10.7759/cureus.75764

**Published:** 2024-12-15

**Authors:** Muneeza Gul, Nazeer Ahmad, Imran Tajik, Asma Javaid, Al Mamoon Khan

**Affiliations:** 1 Orthodontics, Sardar Begum Dental College and Hospital, Gandhara University, Peshawar, PAK

**Keywords:** body mass index (bmi), diet, dietary habits, orthodontics, orthodontic tooth movement, orthodontic treatment, orthodontic treatment and risks, pain, weight loss, weight reduction

## Abstract

Background

Orthodontic treatment, while primarily focusing on correcting dental alignment and occlusion, has been increasingly validated for its potential impact on broader aspects of oral health and general well-being: its potential influence on body weight. While the mechanical effects of orthodontic appliances are well documented in the literature, their potential behavioral impact on weight loss remains underexplored. Beyond its primary role in correcting dental alignment, our study has unveiled a lesser-known benefit: its potential to aid in weight reduction among individuals who have already struggled through conventional methods.

Orthodontic treatment is a surprising yet successful intervention in the field of weight management. Often known only for its use in correcting dental malalignments, our research has revealed an additional benefit: it can help people who have struggled to do so with other conventional methods lose weight. Orthodontic treatment accomplishes the same goals more quickly and with few side effects.

In addition to highlighting the aesthetic and functional benefits of orthodontics, this growing body of knowledge also presents orthodontics as a novel solution for individuals struggling with weight issues. In exploring the mutually beneficial relationship between orthodontic care and weight loss, we looked at a way to achieve both improved overall health and fitness along with dental alignment. Since obesity is associated with a notably higher risk of negative health outcomes, losing weight not only helps you feel better about yourself but also improves your general health and wellness.

This study explores the relationship between orthodontic treatment and reduction in weight among patients undergoing treatment at Sardar Begum Dental College. Its primary objective is to assess and compare changes in body weight among the said patients.

Methodology

This observational prospective study was carried out at the Department of Orthodontics and Dentofacial Orthopaedics, Sardar Begum Dental College, Peshawar. Every patient gave their informed consent and expressed their willingness for participation in the study. An online sample size calculator (OpenEpi) was used. The confidence level was set to 95% with a margin of error of ±5%, and the sample size calculated was 150. The patients were chosen using a convenient sampling technique. The patients' weights were recorded on the days of bonding (T0) and one month (T1), two months (T2), and three months (T3) following bonding. Data was processed using IBM SPSS Statistics for Windows, Version 22.0 (Released 2013; IBM Corp., Armonk, New York, United States). Since the data was not normally distributed, the Wilcoxon signed-rank test was applied.

Results

The Wilcoxon signed-rank test revealed that body weight was significantly reduced one month, two months, and three months after bonding. The body weight on the day of bonding (median=48.200; n=150) was compared to the body weight after one month (median=47.800; n=150), resulting in a statistically significant decrease (z=-9.480; p=0.000) with a large effect size (r=-0.547). The analysis shows a significant decrease in body weight after one month of orthodontic bonding, with a large effect size indicating a substantial impact of the orthodontic treatment on body weight.

Conclusion

Fixed orthodontic treatment appears to have an impact on body weight, i.e., weight is reduced after the first, second, and third months of bonding.

## Introduction

In recent years, the field of orthodontics has expanded its focus beyond the mere alignment of teeth to encompass broader aspects of health and well-being. The goal of orthodontic therapy is to achieve a tooth alignment that is both aesthetically pleasing and functionally efficient [1]. Orthodontic treatment has been extensively studied for its effects on aesthetics, psychological well-being, and overall quality of life. Aesthetic considerations are often the primary motivation for individuals seeking orthodontic care, as they view dentofacial appearance as a crucial factor influencing their overall physical attractiveness [2]. Although orthodontic treatment has historically been thought of as a corrective procedure for dental alignment, its impact on diet, oral health, and the patient's confidence highlights its potential as an additional tool to support and encourage weight management objectives [3]. In addition to its impact on aesthetics, the connection between orthodontic treatment and body mass index (BMI) or weight loss is less commonly examined, yet it remains an area of interest. Some studies indicate that enhanced dental appearance may encourage healthier lifestyle choices, such as improved dietary habits, which could indirectly affect weight management [4,5]. Orthodontic therapy is becoming more widely acknowledged for its positive effects on wider health outcomes, such as regulating your weight, in addition to its dental benefits [6]. This combined effect demonstrates the significance of orthodontic treatment for overall well-being as well as self-esteem by presenting it as both a dental alignment procedure and a possible stimulus and motivation for weight loss, especially for those who have failed to reduce their weight otherwise. The link between malocclusion and self-esteem is well established, with numerous studies showing that improvements in dental aesthetics often result in increased self-confidence and better social interactions [7]. This is especially significant for adolescents, who are becoming more conscious of aesthetic ideals and may pursue orthodontic treatment even in the absence of functional concerns [8,9].

Although there is still more to learn about the association between orthodontic treatment and weight loss, our study is one of these preliminary studies that points towards a broader perspective of orthodontics. Patients may come to see an orthodontist with the hope of getting a beautiful smile and, in parallel, losing weight during the process without any extra effort being put in specifically for weight loss. These two results would motivate patients who not only are driven by the orthodontic benefits but also want weight loss over the course of their orthodontic treatment. The newly found benefit results from the physiological changes induced by orthodontic appliances. Teeth are continuously moved by braces or aligners as they are progressively brought into good alignment. Resultantly, patients frequently feel sensitive or uncomfortable for a short while, especially following dental activations. This procedure can affect eating habits in addition to enhancing oral function and appearance. People may pick softer, healthier, and lower-calorie items over harder, higher-calorie ones as a result of this pain. As a result, switching from foods that are hard to chew to ones that are softer could result in a decrease in total calorie intake [10]. Furthermore, the orthodontic appliance adjustment time usually promotes more deliberate chewing and slower eating. This mindful eating style may help with weight management attempts because it has been linked to lower food intake and better control of hunger signals. Together, these comprehensive methods can support both weight control and general well-being, apart from the most sought-after response, such as dental alignment.

This unanticipated finding of our study raises the possibility of an association between orthodontic treatments and weight loss, which could have been possibly mediated by modifications of eating patterns, such as preferring a soft diet because of pain. This may finally reduce the total calorie intake and eventually lead to a loss of weight. Correcting tooth malalignments provides advantages beyond a beautiful smile, even though the exact mechanisms relating orthodontic therapy to weight loss need to be further investigated. The benefits of orthodontic treatment may become more widely acknowledged as research develops, not just for its functional and aesthetic benefits but also for its possible contribution to weight loss [11]. Although additional investigation is necessary to better understand the exact processes that drive these results and validate the connection between orthodontic treatment and weight loss, our study offers novel and fascinating perspectives on the wider health effects of orthodontic treatment that go outside its regular and conventional focus. Our study provides an intriguing area for the researchers to further investigate and validate these newly found results in orthodontics. Overall, the literature emphasizes that orthodontic treatment extends beyond the correction of dental misalignments, playing a vital role in improving both aesthetic appearance and psychological well-being. The diverse benefits of orthodontic care highlight the significance of incorporating aesthetic and psychosocial considerations into treatment planning and patient counseling [12]. 

Aligned teeth improve chewing efficiency and digestion by enhancing oral function, leading to better nutrient absorption as well as less bloating and discomfort. Furthermore, people may also be more inclined to adopt healthy lifestyle habits, such as eating a balanced diet. In fact, mindful eating is a highly effective way to consume fewer calories which can help with weight management. All things considered, orthodontic treatment can improve general health and dental health [13]. 

## Materials and methods

This study included patients seeking orthodontic treatment at the Department of Orthodontics and Dentofacial Orthopaedics, Sardar Begum Dental College, Peshawar, and was carried out with permission from the Ethical Committee of Gandhara University, with reference number GU/Ethical Committee/2024/144. Patients were recruited by a convenient sampling technique. The sample size was calculated using OpenEpi, with a statistical power of 80%. Based on previous studies, an anticipated small to moderate effect size (Pearson's r) of 0.3 was considered for weight changes. Using a predefined alpha level of 0.05, it was calculated that a sample of 130 participants was required. To account for potential dropouts or loss to follow-up, 150 participants were enrolled. We acquired informed consent from each individual. Over the course of the three and a half months, 150 patients of both genders, ages 16-30, who were either receiving or having comprehensive orthodontic treatment for the first time were enrolled. Prior to the start of orthodontic treatment, baseline measurements (T0) were taken as part of this observational prospective cohort study. After the fixed orthodontic appliances with conventional metal brackets were bonded, follow-up assessments were carried out one, two, and three months after the first treatment (T1, T2, and T3), respectively. To track any weight changes that might be a result of the orthodontic therapy, a weight assessment was part of every examination. Participants had to meet the age requirements of 16-30 years of age, with any kind of malocclusion, and be undergoing their first orthodontic treatment in order to be considered for inclusion. Athletes, people enrolled in gyms or fitness clubs, people with a history of prior orthodontic treatment, people with systemic illnesses, disabilities, or stress, patients on long-term obesity medications (e.g., sibutramine, orlistat), people undergoing steroid therapy, and people on a weight-loss diet were all excluded. Data was analyzed using IBM SPSS Statistics for Windows, Version 22.0 (Released 2013; IBM Corp., Armonk, New York, United States). Following the Anderson-Darling normality test, a non-parametric Wilcoxon signed-rank test was employed to investigate changes in weight, as the data did not follow a normal distribution. The Wilcoxon signed-rank test has been used to detect the pre- and post-treatment weights of the participants. The purpose of this test was to evaluate the participants' weight before and after therapy. Changes in body weight were the study's main end measure. 

## Results

A total of 150 individuals were included in the study, ranging from 16 to 30 years of age. Out of them, 61 were males (mean age: 22±3) and 89 were females (mean age: 22±3) (Table 1 and Table 2).

**Table 1:** Descriptive analysis of gender-based distribution of age

Gender	Mean	Median	Mode	Standard deviation
Male	22	21	19	3
Female	22	21	18	3
Total	22	21	19	3

**Table 2:** Gender-based demographics of the participants

		Frequency	Percent
Gender	Male	61	40.7%
	Female	89	59.3%
	Total	150	100%

**Figure 1:**
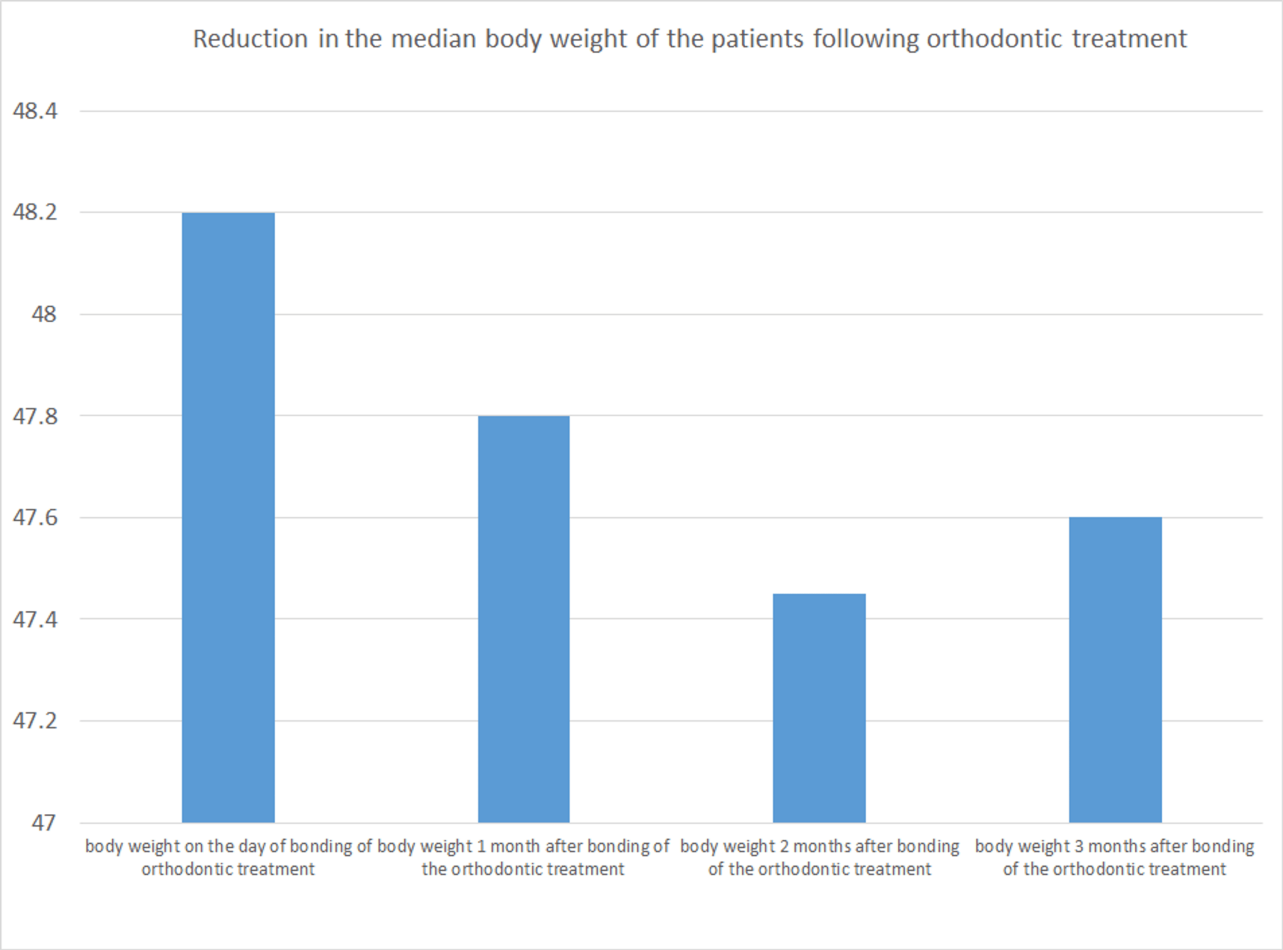
Distribution of weight loss over the course of the orthodontic treatment

The results of the Wilcoxon signed-rank test showed that there were notable variations in body weight over time. Body weight decreased from day 1 to month 1 (Z=-9.480; p<0.001), from day 1 to month 2 (Z=-9.713; p<0.001), and from day 1 to month 3 (Z=-9.952; p<0.001). This decrease was statistically significant. Subsequent analyses revealed a noteworthy decline between the first and second months (Z=-9.952; p<0.001), as well as between the second and third months (Z=-8.556; p<0.001). These results indicate a consistent reduction in body weight over the three months (Table 3 and Figure 1).

**Table 3:** Pre- and post-treatment comparison of weight P-value less than 0.05 was considered significant

Comparison	N	Median body weight (day 1) T0	Median body weight (post-treatment)	Z score	P-value	Effect size	Interpretation
Body weight after month 1 vs. day 1	150	48.200	47.800	-9.480	0.000	-0.547	Significant decrease in body weight after the first month
Body weight after month 2 vs. day 1	150	48.200	47.450	-10.205	0.000	-0.5891	Significant decrease in body weight after the second month
Body weight after month 3 vs. day 1	150	48.200	47.600	-9.713	0.000	-0.5607	Significant decrease in body weight after the third month
Body weight after month 2 vs. month 1	150	47.800	47.450	-9.520	0.000	-0.77	Significant decrease in body weight from the first to the second months
Body weight after month 3 vs. month 2	150	47.450	47.600	-8.556	0.000	-0.69	Slight increase in body weight from the second to the third months

## Discussion

The objective of this study is to investigate the relationship between orthodontic treatment and weight loss, focusing on changes in dietary habits associated with the treatment. It aims to analyze the impact of these dietary changes on body weight during and after orthodontic treatment and to determine the extent to which orthodontic treatment influences long-term weight management. The findings revealed that the body weight was reduced after the first, second, and third months of treatment. The current study was carried out at Sardar Begum Dental College in Peshawar, in the Department of Orthodontics and Dentofacial Orthopaedics. The study's sample size comprised 150 participants, aged between 16 and 30 years. The pre-treatment weight of the patients who were not treated yet was obtained on the day of bonding (T0). Then after one month (T1), two months (T2), and three months (T3), their weights were recorded. The body weight on the day of bonding (median=48.200; n=150) was compared to the body weight after one month (median=47.800; n=150), two months (median=47.450; n=150), and three months (median=47.600; n=150), showing a pattern of initial weight reduction followed by stabilization. The findings indicate an initial decrease in body weight within the first two months following orthodontic bonding, likely due to changes in dietary habits. By the third month, the weight begins to stabilize, suggesting that patients may adapt to their new eating patterns over time. This highlights the impact of orthodontic treatment on body weight, with potential implications for nutritional guidance during this period. The median value has reduced indicating a very gradual and a very healthy weight loss.

Another interesting study finding suggests that after a year, 43.4% of patients had a drop in their BMI, 45.8% saw a slight to moderate increase, and 10.8% of patients saw no change in their BMI. There was no statistically significant difference [14]. It has been demonstrated that orthodontic treatment has a major impact on patients' food habits and, as a result, body weight. The discomfort of the orthodontic treatment, dietary limits prescribed by orthodontists, and the adjustment to new eating habits brought on by the orthodontic appliances are all possible causes of the dietary alterations. First of all, a lot of patients change their eating habits during the early phases of orthodontic treatment due to pain and discomfort. Negruțiu et al. observe that more discomfort is reported by adult patients, which may lead to a decrease in food intake and, eventually, weight loss [15]. Similarly, Ajwa et al. point out that patients may avoid hard or sticky meals that could make their pain worse if they are advised to eat soft foods to reduce discomfort. This can result in major changes to dietary patterns [16]. This is consistent with research by Kılınç and Sayar, who found that these dietary adjustments cause many patients to lose weight during the first three months of treatment [17]. Additionally, Sandeep and colleagues warn that the stress brought on by orthodontic treatment might raise nutrient use, upping the patients' nutritional needs, which might not be satisfied if they are consuming less food [18].

Orthodontic patients often experience not only discomfort but also specific dietary recommendations from their orthodontists, which might further alter their eating habits. According to Aljohani and Alsaggaf, patients who have to clean their teeth more regularly to get rid of food particles stuck in their gadgets may decide to eat less frequently in order to preserve oral hygiene [19]. Shalchi et al. reported that following the recommendations of orthodontists, a soft diet might result in a reduction of caloric intake and possible weight loss [20]. Furthermore, Johal et al. discovered that patients frequently anticipate having their food intake restricted as a result of orthodontic treatment, which may result in additional dietary restrictions [21]. It's important to take the psychological effects of food modifications into account. As they become more aware of their food choices, patients frequently report feeling as though their eating habits have improved while undergoing treatment [22]. This change can result in better eating habits, but if the intake of calories is not sufficient to fulfill their nutritional demands, it can also cause unintended weight loss. de Couto Nascimento et al. point out that eating a balanced diet is essential for good health in general and for the orthodontic treatment to work since poor nutrition might alter the way the tissue reacts to the forces used during treatment [23].

The current study's findings on the impact of orthodontic treatment on body weight due to changes in dietary habits align with existing research on the treatment's effects on quality of life and self-esteem. While studies have reported initial declines in oral health-related quality of life (OHRQoL), they also show eventual improvements in self-esteem and quality of life. Additionally, research indicates that orthodontic therapy enhances OHRQoL. This study builds upon these findings by exploring the treatment's impact on body weight and dietary habits. Interestingly, the observed weight loss and reduced BMI in patients undergoing orthognathic surgeries may be related to the current study's findings, suggesting a potential link between orthodontic treatment, dietary habits, and body weight. Similarly, another study's findings suggest that during the initial three months of fixed orthodontic therapy, there was a negative effect on overall OHRQoL. However, this impact diminished over time, and OHRQoL scores eventually returned to pre-treatment levels. Additionally, there was a notable improvement in self-esteem as a result of the treatment [5]. On the other side, this study claims that orthodontic treatment leads to a substantial boost in self-esteem and quality of life, offering psychological advantages for adult patients undergoing oral rehabilitation [2]. Another study of the same nature by Settineri and his colleagues finds that OHRQoL improves during orthodontic therapy [24]. One study suggests that orthognathic surgeries, both monomaxillary and bimaxillary, also resulted in weight loss and reduced BMI [25]. Another similar study found that patients with elevated BMI need extra consideration during orthodontic therapy because an elevated BMI appears to be a risk factor for decreased cooperation, a longer treatment period, and more oral health-related issues throughout multi-bracket treatment [26].

However, more studies with larger samples may be necessary to confirm and validate these results. Also, more studies with longer periods of follow-up are required. Even though there were 150 participants, the results might not apply to people who are having orthodontic treatment for reasons other than those listed in the age range of 16-30 years. Longer follow-up may be required for a more thorough understanding, since the relatively short duration of three months may not capture the long-term effects of orthodontic treatment on body weight. One limitation of this study is the lack of assessment of the participants' BMI, which could provide a more comprehensive understanding of changes in body composition during orthodontic treatment. Without incorporating BMI measurements, the study may not fully capture the potential effects of orthodontic treatment on overall body composition, which could limit the interpretation of weight changes observed in the participants. Furthermore, the stringent exclusion criteria, which included conditions pertaining to stress, systemic disorders, and particular medication, may have reduced the number of participants, which could have limited how broadly the findings could be applied. Weight measurements were the only metrics utilized in the study to track changes, which may not accurately reflect changes in body composition or other physical health parameters. Uncontrolled external factors, including dietary habits, physical activity levels, and other lifestyle modifications, may have an independent impact on weight changes from orthodontic treatment. Data consistency may also be impacted by individual perception differences and treatment procedure adherence. Furthermore, compared to parametric tests, the use of non-parametric tests may be less effective at identifying minute changes, even while they are acceptable for the data distribution. These elements should be taken into account when interpreting the study's results and point out areas in which more research may shed light in the future. 

## Conclusions

This extensive study found that patients' weight significantly decreased while receiving orthodontic therapy. Through rigorous observation of a cohort of patients at different phases of their orthodontic treatment, the study discovered that weight loss was uniform among the cohort. There were several causes that led to this result. Patients with braces or aligners generally avoided specific foods that could cause discomfort or break their appliances and, as a result, ate less. Patients also mentioned that their eating habits had changed, favoring softer, lower-calorie foods that were simpler to eat while wearing orthodontic appliances. Changes in eating habits and weight may also have been influenced by psychological variables, such as elevated self-consciousness about appearance and a greater emphasis on personal health. Also, this study emphasizes how crucial it is to keep an eye on your weight and food intake and recommends that orthodontists take these possible side effects into account while organizing and managing patient care throughout orthodontic treatment.
